# Generation of Germ-Free *Ciona intestinalis* for Studies of Gut-Microbe Interactions

**DOI:** 10.3389/fmicb.2016.02092

**Published:** 2016-12-27

**Authors:** Brittany A. Leigh, Assunta Liberti, Larry J. Dishaw

**Affiliations:** ^1^College of Marine Science, University of South Florida (USF)St. Petersburg, FL, USA; ^2^Department of Pediatrics, College of Medicine, University of South Florida (USF)St. Petersburg, FL, USA

**Keywords:** germ-free, *Ciona intestinalis*, Gut colonization, microbiome, host-microbe interaction

## Abstract

Microbes associate with animal hosts, often providing shelter in a nutrient-rich environment. The gut, however, can be a harsh environment with members of the microbiome settling in distinct niches resulting in more stable, adherent biofilms. These diverse communities can provide orders of magnitude more gene products than the host genome; selection and maintenance of a functionally relevant and useful microbiome is now recognized to be an essential component of homeostasis. Germ-free (GF) model systems allow dissection of host-microbe interactions in a simple and direct way where each member of the symbiosis can be studied in isolation. In addition, because immune defenses in the gut are often naïve in GF animals, host immune recognition and responses during the process of colonization can be studied. *Ciona intestinalis*, a basal chordate, is a well-characterized developmental model system and holds promise for addressing some of these important questions. With transparent juveniles, *Ciona* can be exposed to distinct bacterial isolates by inoculating GF artificial seawater; concentrated bacteria can subsequently be visualized *in vivo* if fluorescent stains are utilized. Rearing GF *Ciona* is a first step in untangling the complex dialogue between bacteria and innate immunity during colonization.

## Introduction

The ability to establish germ-free (GF) versions of both traditional and non-traditional model systems has become essential in studies that aim to develop and expand our understanding of the role of host-associated microbial communities in both health and disease. Because of diverse phylogenetic histories, each of these models can offer insight into the role microbes have played in shaping molecular and ecological processes that sustain homeostasis at host mucosal tissue surfaces. The gut, in particular, is a highly dynamic ecosystem where homeostasis is imperative to maintaining host health (Round and Mazmanian, 2009; Tlaskalová-Hogenová et al., 2011). The initial establishment of these microbes early in development influences long-term community structure and shapes host response (Nicholson et al., 2012; Sommer and Backhed, 2013).

The sea squirt, *Ciona intestinalis*, is a marine protochordate; these invertebrates (Subphylum: Tunicata) are particularly interesting because they represent the most basal chordate condition (Dunn et al., 2008), yet possess a gut characterized by the same three main anatomic compartments found in the more recently diverged vertebrates: esophagus, stomach and intestine. During its life cycle, the embryo develops into a swimming tadpole larva that attaches to a substrate and undergoes metamorphosis, losing its notochord and becoming a sessile adult (Chiba et al., 2004). In the early stages of metamorphosis, the intestine disc (gut primordium) begins to differentiate into its anatomical compartments and, at stage 4 of the 1st ascidian juvenile, the digestive tract opens to the external environment, initiating the process of feeding and microbial colonization. Moreover, *Ciona* possesses an immune system that relies exclusively on what is known as innate immunity; adaptive immunity, and the signature genes associated with antigen recognition, antibody production, and memory, is restricted to the vertebrates (Azumi et al., 2003). Thus, this model allows investigators to utilize a chordate model system while focusing attention on defining the role(s) of innate immunity during microbial colonization of gut epithelial surfaces.

The protocol described herein was developed by modifying previously described methods to rear germ-free zebrafish (Pham et al., 2008). In *Ciona*, a single fertilization can give rise to hundreds of juveniles, which can be reared easily at the bench-top, facilitating numerous experimental conditions and/or replicates. Briefly, *Ciona* is fertilized *in vitro*, and within the first hour of development the zygotes are treated to remove or kill microorganisms living on or within the chorion. Subsequently, the embryos, and later the juveniles, are reared in artificial seawater (ASW) supplemented with antibiotics and a simple antifungal solution. One week after fertilization, when the animals reach stage 4 of metamorphosis, the antibiotics and antifungal solution are removed, and the freshly changed water can then be inoculated with one (monoassociation) or more (mixed community) microbes of choice for colonization experiments. The *Ciona* juveniles will continuously siphon and concentrate microoganisms in its gut where a selection process likely occurs. Because *Ciona* juveniles are also transparent, visual detection and localization of fluorescently labeled bacteria facilitates visualizing the process.

## Materials and equipment

**Figure 1:**
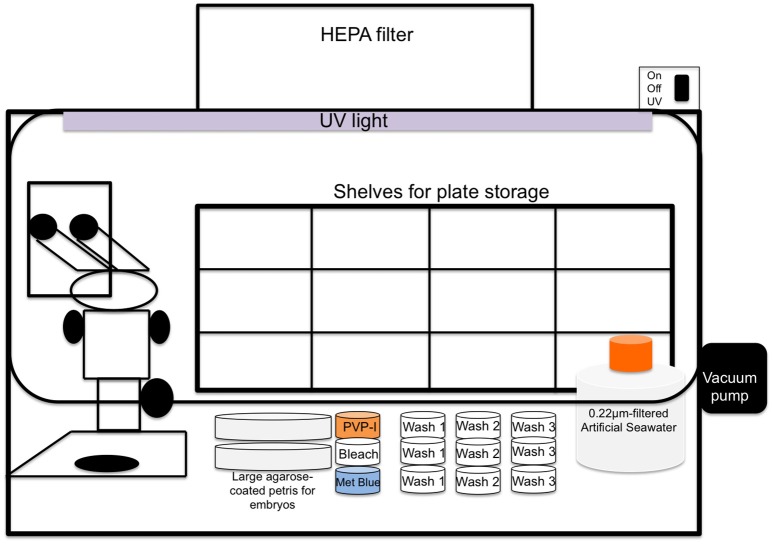
**Schematic illustration of cleanroom with incorporated stereoscope and layout of other materials;** cleanroom should have the ability to maintain positive pressure by HEPA filtration while in use and a UV light should be available to help sterilize environment between experiments.

**Bench-top cleanroom fitted with HEPA-filter ventilation (two separate cleanrooms are optimal but not required; available positive pressure/laminar flow is ideal as long as it can be turned on/off to control evaporation) as depicted in Figure 1**

**(The following should be within or near the cleanroom)**

- pipets and tips
- automatic pipet
- large waste beaker
- serological pipets
- labeling marker
- 50 mL and microcentrifuge tube rack
- 500 mL or 1 L bottle top filters (0.22 μm)
- vacuum pump
- stereo microscope or equivalent
- 10 mL syringes for filtering smaller volumes
- 0.22 μm syringe filters

**Additional equipment:**

- refractometer
- sterile petri dishes (150, 100, and 60 mm)
- 50 and 15 mL conical tubes
- 70 μm sterile cell strainer baskets (Corning #431751)
- centrifuge
- autoclave
- thermocycler
- lab coat
- disposable gloves
- acid washed glass bottles

**Reagents:**

- Instant Ocean® (or equivalent artificial salt mix)
- 10% (wt/vol) polyvinyl pyrrolidone-iodine complex (PVP-I)
- 5% (vol/vol) bleach stock solution
- Methylene blue, 1% (LabChem LC19940-7)
- 70% isopropanol
- 1% agarose LE (Fisher BMA50004; gel electrophoresis grade) in ASW
- Pen/Strep 100x stock (Fisher BP295950)
- ZymoResearch Tissue & Insect DNA MiniPrep (Zymo Research, Irvine, CA, USA)
- Bacterial 16S rRNA PCR primers (27F and 1492R)
- Fungal Internal Transcribed Spacer (ITS) 18S primers (ITS1 and ITS4)(White et al., 1990)
- *Ciona intestinalis* cytoskeletal actin (gene accession number AJ297725) PCR primers, designed in-house (F5′- ATGGACGATGATGTTGCCG, R5′- TTAGAAGCATTTGCGGTGGAC)
- 2X Master Mix, premade commercial mix (Preferred: Promega PCR Master Mix M7505)
- Preferred media and culture plates for sterility checks (Preferred bacterial media: Marine Broth (Fisher DF0791-17-4); Nutrient Broth +Sea Salts (Fisher DF0003-07-0); Brain-Heart Infusion Broth +Sea Salts (Fisher DF0037-15-0); Tryptic Soy Broth +Sea Salt (Fisher DF0369-15-8); Sea Salts (Sigma S9883) added at approximately 40 g/L where required, adjusting concentration to minimize precipitation)

**Stepwise Procedures**

**A) Reagents setup**

(1) *Preparing and maintaining a germ-free environment*
  All decontamination and subsequent maintenance of GF juveniles under aseptic conditions occurs within the confines of a bench-top cleanroom equipped with a HEPA filter that has been thoroughly sanitized and UV-treated prior to starting any of the procedures. To help maintain aseptic conditions in the cleanroom, all activities are performed while wearing disposable lab coats with tight, elastic, closures at wrists, and disposable nitrile gloves that fit over the elastic fitted sleeve. Before entering the cleanroom environment, gloved hands, and any other object, should be rigorously cleaned by rubbing in the presence of 70% isopropanol.
(2) *ASW preparation*
  ASW working solution should be prepared, using Instant Ocean® or equivalent, at least 2 days prior to fertilization to allow salts to equilibrate. *Optional: while it as advised that one always follow manufacturer's recommendations, saturated salt solutions can be made as long as the final target salinity, after dilution, is approximately 33 parts per thousand (ppt)*. Filter the ASW through a 0.45 μm filter to remove large particulates and autoclave; because of the potential for evaporation during the autoclaving process, the final salinity must be confirmed with small aliquots using a refractometer. Be sure to use acid washed bottles to prepare and store ASW as any remnants of soap will reduce fertilization rates. The ASW is then stored at room temperature until use. All instances below that utilize ASW refer to this sterilized version.
(2.1) Before use, the ASW is filter-sterilized using 0.22 μm- bottle-top filters within the cleanroom environment. The prepared ASW remains in the cleanroom.
(3) *Dish preparation for larval development- timing 40 min*
  To generate and maintain GF animals, sterilization steps are included that alter chorion integrity; embryos with disrupted chorions can stick to plastic dishes, a process that can significantly decrease viability. To keep the developing embryos from sticking to the plastic dishes, the plates are coated with a thin layer of sterilized 1% agarose. Once the larvae hatch, animals are transferred to non-coated/non-treated dishes for attachment and metamorphosis. Agarose is dissolved in ASW to a final concentration of 1% and autoclaved (it is not critical that the agarose remain at exactly 1%). In the cleanroom, large petri dishes (150 mm) are coated with a thin layer of 1% agarose by pouring a small amount and tilting plate to cover. Generally, two dishes are sufficient for plating one batch of fertilized eggs. The lid is replaced and plates are dried, upside down, in the cleanroom for about 20 min. Plates should be prepared not more than 1 h prior to fertilization since the thin layer of agarose will dry out quickly.
(4) *Preparation of sterilization solutions—timing 15–30 min*
(4.1) **Antibiotic ASW**: 1L of ASW supplemented with 1x final concentration of Pen/Strep and 10 μL of 0.1% Methylene blue is prepared and filter-sterilized using a 0.22 μm filter within the cleanroom. The solution is aliquotted (50 mL each) with a serological pipet into two large 1% agarose-coated petri dishes.
(4.2) **0.1% PVP-I solution**: 10 mL of 0.1% PVP-I working solution in ASW is prepared through serial dilution from a stock solution. The solution is filter-sterilized using a 0.22 μm filter and 10 mL syringe into a small petri dish (60 mm) within the cleanroom. Three additional dishes (60 mm) with 10 mL ASW each are prepared for subsequent wash steps.
(4.3) **0.003% bleach solution**: 10 mL of a 0.003% bleach working solution is prepared in ASW through serial dilutions from a stock solution. The solution is filter-sterilized using a 0.22 μm filter and 10 mL syringe into a small petri dish (60 mm) within the cleanroom. Three additional dishes (60 mm) with 10 mL ASW each are prepared for subsequent wash steps.
(4.4) **0.001% methylene blue solution**: 10 mL of a 0.001% methylene blue working solution is prepared in ASW through serial dilution from a stock solution. The solution is filter-sterilized using a 0.22 μm filter and 10 mL syringe into a small petri dish (60 mm) within the cleanroom. An additional dish (60 mm) with 10 mL ASW is prepared for the final wash step.

**B) Fertilization and sterilization protocol**

From here onwards, there are no stopping points within the protocol.

**Figure 2:**
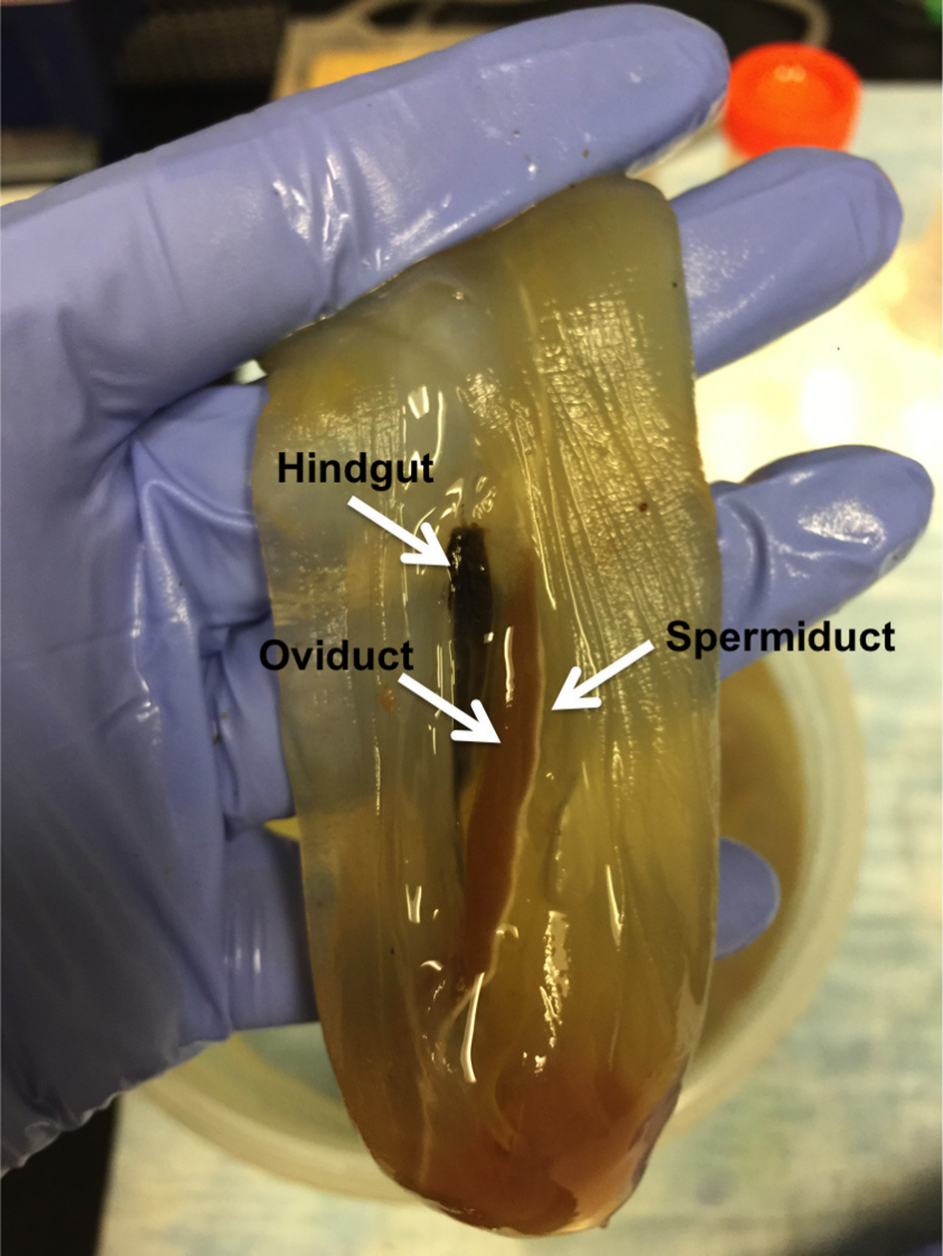
**Anatomy of the ***Ciona intestinalis*** reproductive organs**. The animals, once opened, show the gut, oviduct and spermiduct running parallel to each other along *Ciona* body axis.

(5) *Fertilization procedure—timing 15–30 min The isolation and fertilization of eggs is performed on the bench-top outside of the cleanroom*.
  Adult animals, harvested from the wild, arrive in the lab and are held in ASW until *in vitro* fertilization is performed. Individual animals, which are hermaphroditic, are surgically dissected for eggs and sperm collection.
(5.1) Freshly harvested *Ciona* with visibly mature gametes (eggs are reddish and high in the oviduct) are placed on their sides in a new plastic dish. The outside of the animal tunic is wiped gently with iodine and alcohol solutions, and the tunic is opened with a fresh scalpel to expose the inside near the oviduct and spermiduct, which run parallel to the intestines (Figure 2). Under the tunic, a thin, clear, membrane must be torn to expose the oviduct.
(5.2) Five to ten animals are used for extraction of gametes. The top of the oviduct is pierced with sterile tweezers, and using a Pasteur pipet the eggs are extracted and deposited into a 50 mL conical tube containing 40 mL ASW. Sperm are collected, preferably with the least amount of water possible to keep them inactivated and concentrated, and pooled in a 1.5 mL tube.
(5.3) The egg mixture (in a 50 mL conical) is then fertilized by adding a small amount of sperm mix (1–2 drops) and placed on a rotating wheel to allow adequate mixing. After 5 min, the fertilized eggs are strained through a sterile 70 μm basket to remove excess sperm and washed in ASW.
(5.4) Some fertilized eggs are separated into a small petri dish on the bench-top to serve as a fertilization control, prior to starting the sterilization procedure. Monitoring the development of these embryos is important for evaluation of fertilization success and percent viability; it is also an important control for the sterilization procedure, which can impact embryo viability.
(6) *Sterilization procedure- timing 30–35 min*
  The entire sterilization process should be finished before the eggs reach the two-cell stage, approximately 50 min from the moment of fertilization in step (5.2). This time frame is essential because the damage to the chorion inflicted by the sterilization procedure makes the embryos particularly fragile. Hence, it is important to plate them before the advancement of cleavage to avoid excessive manipulation during these critical phases of development.

**Figure 3:**
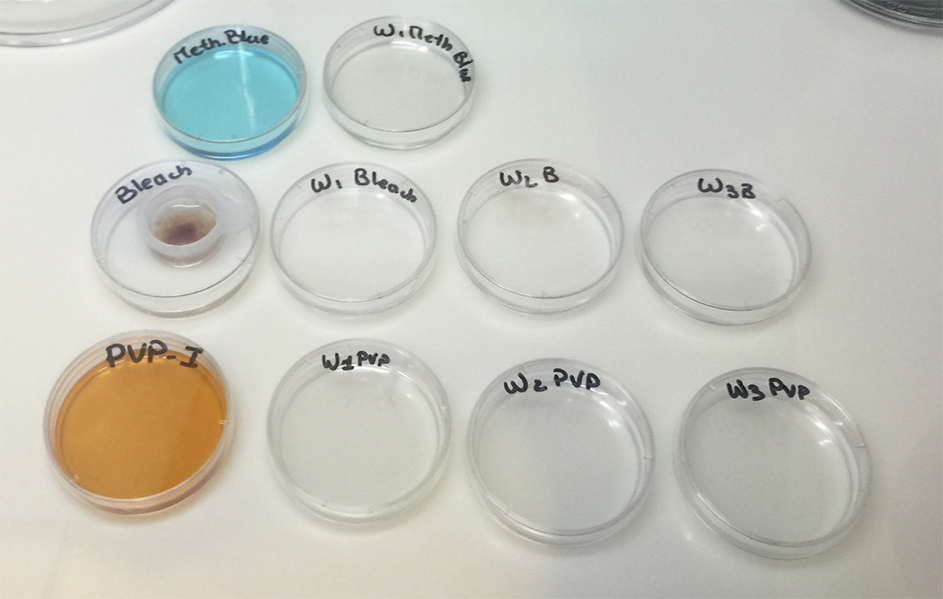
**Embryo handling during sterilization procedure**. Once eggs are fertilized, they are placed into a 70 μm basket for handling. Embryos remain in this basket to facilitate transfer between solutions.

**Figure 4:**
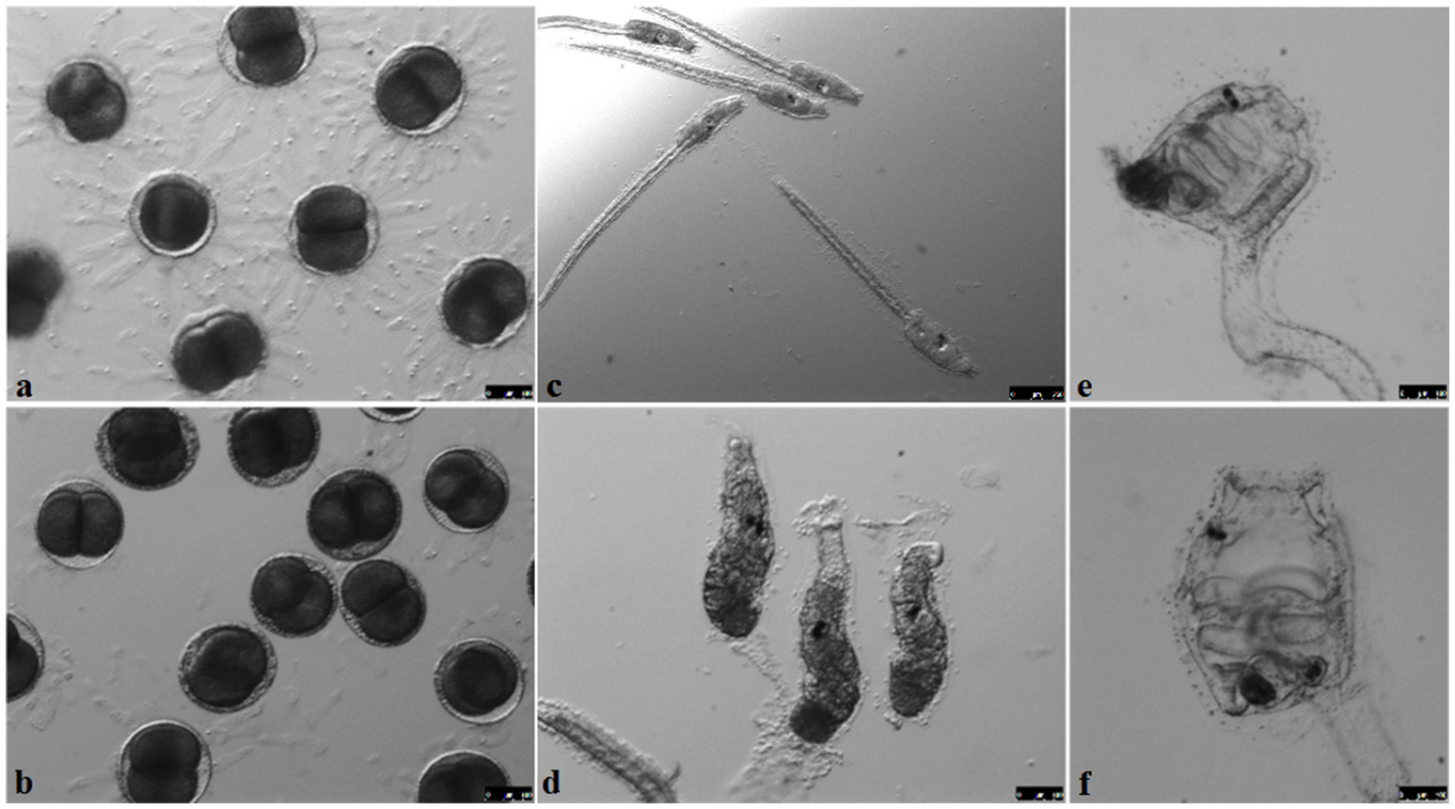
**Developmental stages of GF ***Ciona intestinalis*****. Eggs before **(A)** and after **(B)** sterilization show a compromised chorion. The development of juveniles after fertilization **(C–F)** is similar to conventionally-reared animals described previously in FABA developmental tables. Scale bars: 100 μm; **(C)** 250 μm.

During the entire sterilization procedure, the fertilized eggs are maintained in a 70 μm basket to facilitate solution changes (Figure 3).

(6.1) The fertilized eggs are gently submersed into 0.1% PVP-I solution for 2 min, minimizing air bubble formation; any longer at this step, and viability of the embryos drops significantly. Embryos are washed three times in ASW, agitating lightly to thoroughly wash.
(6.2) The fertilized eggs are gently immersed in 0.003% bleach solution for 20 min, agitating the embryos every few minutes and avoiding air bubble formation to ensure that all the embryos are in contact with the solution. The embryos are washed three times in ASW, again agitating gently. After this step, most of the chorions, with follicle cells removed, should be damaged and/or coming loose from the embryos (Figure 4B).
(6.3) The fertilized eggs are gently immersed in 0.001% methylene blue solution for 10–30 s followed by a quick rinse in ASW. This step should effectively treat fungal contamination that may have been carried over by the eggs.
(6.4) Using a 1 mL (or P1000 type) personal pipet with large bore sterile tips, the embryos are gently transferred onto agarose-coated petri dishes containing antibiotic ASW prepared as described above in (3) *Dish preparation for larval development* and (4.1) *Sterilization solution preparation* sections. Minimize any additional disturbance to the plates to prevent shearing of the eggs after the chorions are damaged.
(6.5) Approximately 18 h are allowed for larval development to proceed, until the eggs hatch and release swimming tadpoles.
(7) *Larva settlement-timing 30–45 min*
  Once larvae hatch, they will begin to stick to each other or shed egg debris if plated densely. Thus, one should work quickly to transfer freshly hatched, swimming tadpoles, onto fresh dishes. Larvae that have attached to each other or to debris are difficult to separate and should simply be discarded. However, if larvae are transferred before they hatch or begin swimming, viability again will be significantly compromised.
(7.1) New, *uncoated*, petri dishes (6, 10, or 15 cm) with antibiotic ASW solution (4.1) are prepared.
(7.2) Each dish is labeled to adequately track them for sterility checks and animal development.
(7.3) Swimming larvae are gently transferred with a large bore sterile tip and spread out onto new dishes and allowed to attach overnight. Minimal water is added to ensure the larvae do not attach on the sides of the dishes where they become difficult to observe. Larvae that are not attached by the following morning will be removed from the plates during the first water change.

**C) Verifying and maintaining germ-free animals**

**Figure 5:**
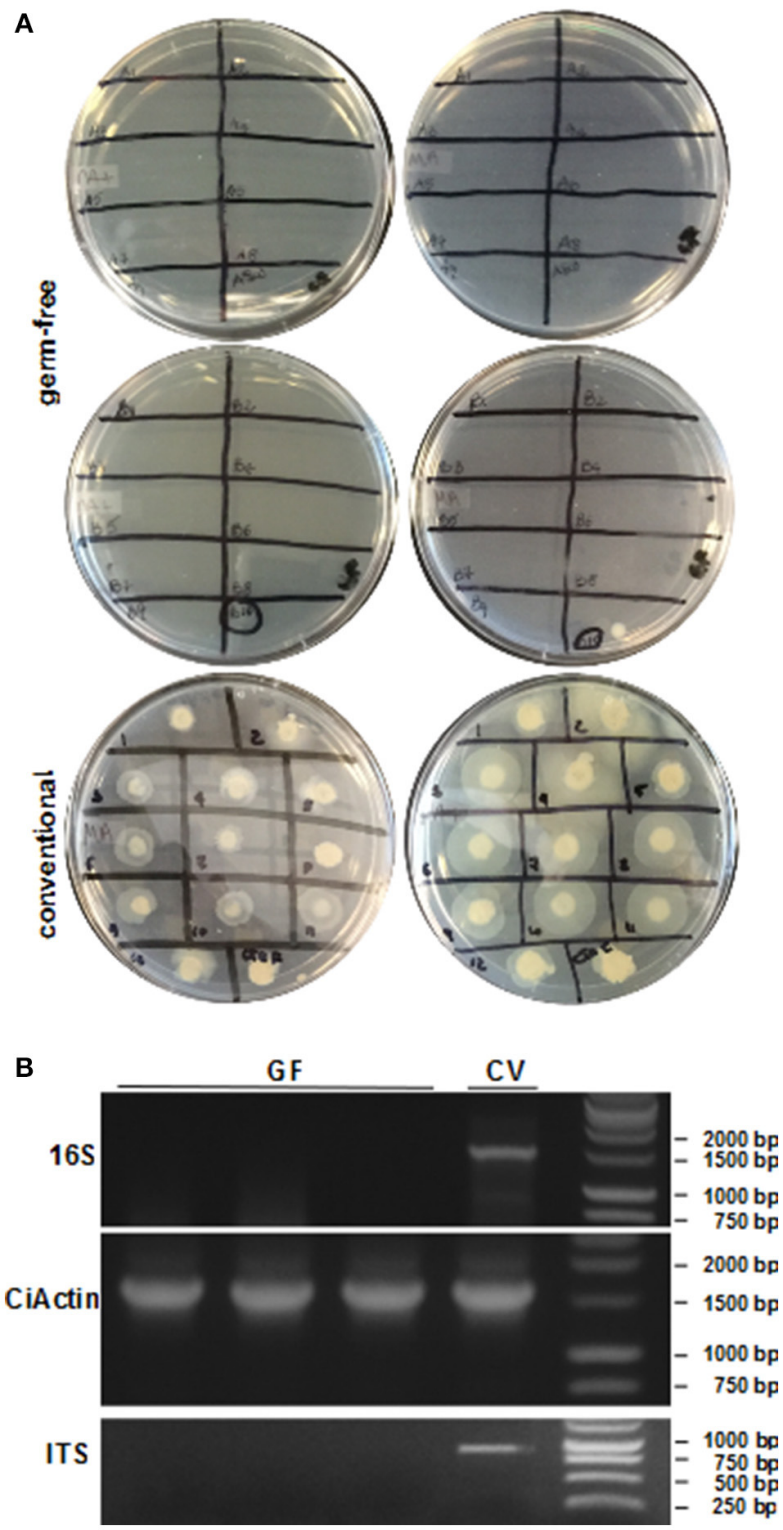
**Sterility tests of the GF procedure. (A)** ASW or FSW from dishes of GF or conventionally-reared animals, respectively, is spotted on two different media plates (left: marine agar, right: nutrient agar + sea salts). Cultures from GF animals (top) show no bacterial growth except for one plate. Water from conventional animals (bottom) shows a diverse microbial community. **(B)** PCR using DNA isolated from GF and conventional juveniles shows no 16S rRNA gene amplification in GF animals. The CiActin gene amplification is used as a control for DNA extraction of the animal. PCR on ASW or FSW using ITS primers demonstrates the presence of fungi in conventional and not in GF dishes.

(8) *ASW Changes and Sterility Tests*
  After larval settlement, ASW is changed every 48 h using aseptic conditions and sterile serological pipets. Waste ASW is collected within the cleanroom, a small aliquot saved for sterility test (see #2 below), and the remainder disposed; all transfers of fluid should be done with careful pipetting technique as one would use in standard cell culture. During the first week of development, animals are maintained in antibiotic ASW; after this period they are transferred to ASW without any supplementation. Before each water change, sterility checks are performed on each individual plate using three different approaches:
1) Spot plating on various media plates (see reagent prep). ASW (10 μL) from each dish is spotted onto a media plate that has been warmed to room temperature (Figure 5A). Some preferred plates are marine agar, nutrient agar + sea salts, brain-heart infusion + sea salts and tryptic soy agar + sea salts. Cultures are maintained at room temperature for a minimum of 72 h, or the duration of the experiment.
2) Pelleting of spent culture media (used ASW) to detect bacteria by PCR. During water changes, spent ASW from each dish is collected into 1.5 mL microcentrifuge tubes and spun at maximum speed for 10 min to pellet any potential contaminant present. The supernatant is replaced with 50 μL of nuclease-free water because salt water inhibits the PCR reaction; the redissolved material becomes template for PCR using the 16S rRNA gene and ITS primers defined above.
3) Total genomic DNA is isolated from randomly sampled animals on each GF dish and used as template for PCR. Before experimental colonization, animals are randomly sampled with a sterile prick and recovered with a large bore pipet tip. Total DNA is isolated using an equivalent DNA extraction kit (the Zymo kit described above works well with our small tissue samples); each plate is done individually to verify that GF animals have remained bacteria-free. The 16S rRNA and actin primers described above are used on the isolated DNA to confirm absence of bacteria and the presence of animal tissue, respectively. Water from the plates can be checked for fungal contamination by plating and by PCR with ITS-specific primers (Figure 5B). Some of the ITS primers cross-amplify host DNA; however, additional ITS primer options exist (Martin and Rygiewicz, 2005).

*Sterility tests are performed every 48 h during development. If all the sterility tests are negative, germ-free animals have been generated and they can be used experimentally for microbial colonization*.

**D) Development and Colonization of Germ-Free Animals**

(9) *Determination of developmental stages*
  Germ-free animals take approximately 4 days to reach stage 4 where the siphons open; subsequently, the animals can begin to filter water. Refer to the FABA developmental table (http://chordate.bpni.bio.keio.ac.jp/faba/1.4/top.html) to determine phenotype for stage 4 animals. Prior to this stage, the gut remains sterile (uncolonized). Plates may develop at different rates depending on animal density; therefore, it is imperative to check every plate for stage 4 animals before beginning exposures.
(10) *Experimental Colonization with Fluorescent bacteria*
(10.1) A freshly isolated colony from a bacterial strain of interest is grown overnight in its preferred culture media and conditions.
(10.2) The optical density (OD_600_) is then measured and adjusted to the desired concentration relative to the final volume of the petri dish to be exposed. Normally 10^6^ cells/mL is well tolerated by the animals (depending on bacterial strain); when the bacteria are labeled with vital dyes at this cell density, they can be visualized if they become concentrated in the gut. The amounts to use can be correctly estimated by OD_600_ once a growth curve is generated; colony-forming units (CFU) of bacterial abundance can be performed at respective intervals.
(10.3) Example vital dye: The bacteria are stained for 15 min using *BacLight* green (Invitrogen Molecular Probes B-35000), as per manufacturer's instructions. Green (Absorbance: 480 nm; Emission: 516 nm) is used as a starting dye because it provides the least amount of background in the live, whole-mount, animals. The bacteria are then washed twice in ASW and resuspended in a final volume of ASW to cover entire petri dish. The culture media (spent ASW) from the GF animal dishes is then replaced according to *ASW Changes and Sterility Tests* section above; in short, the plate is removed from the cleanroom, and the resuspended, labeled bacteria in ASW are added to the culture dishes. The dishes are incubated at room temperate under low light conditions. The animals, when observed under a stereoscope equipped with a fluorescent light source and camera, can be seen to immediately uptake the bacteria and concentrate the signal in their guts. Movement, and presumably settlement of some bacteria, can be visualized along the gut wall within 1 h after exposure begins and also detected in the days that follow.

## Anticipated results and discussion

**Figure 6:**
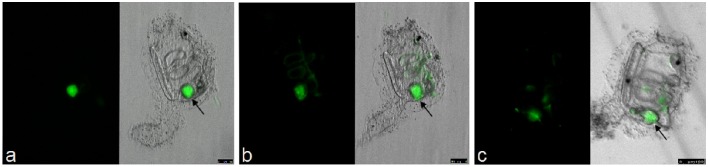
**Bacterial exposure to GF animals**. Bacteria stained with *BacLight* green and then exposed to stage 4 GF juveniles. Three different bacterial strains (**A**. *Vibrio*, **B**. *Pseudoalteromonas*, and **C**. *Shewanella*) were each concentrated within the gut of the animal within 1 h of exposure with little to no detectable signal in any other tissue outside the gut. Scale bars: 100 μm.

Normally, the fertilization controls will have a hatching percentage of approximately 95% during spawning seasons. The eggs maintain their chorion structure with intact follicles as shown in Figure 4A. After sterilization, the chorion becomes damaged, resulting in loss of follicles (Figure 4B) and egg viability drops to approximately 60–70% of embryos hatching. Compared to conventional animals grown in normal seawater, GF embryo development and timing of metamorphosis remains quite similar (Figures 4C–F; compare to *Ciona* developmental tables at FABA1 (http://chordate.bpni.bio.keio.ac.jp/faba/1.4/top.html) and FABA2 (http://chordate.bpni.bio.keio.ac.jp/faba2/2.2/top.html; Hotta et al., 2007). Success in the generation of GF *Ciona* varies between fertilization events and can result in 70–100% sterile animals, but the amount of eggs and time of year are crucial. An example of a successful sterility check is shown in Figure 5. After 3–4 days, animals reach stage 4 of 1st ascidian juvenile, and it is at this stage that the digestive tract opens to the external environment initiating the process of microbial colonization. Animals will remain at stage 4 until they are fed. Similar to previously described observations of developmental delays in zebrafish, GF *Ciona* do not proceed through stage 4 without additional dietary supplementation (Rawls et al., 2004; Semova et al., 2012), e.g., *Nannochloropsis* commercial paste (Cat# PM36N, Pentairaes.com). Once bacteria (or other microbes) are included in the water for colonization experiments, the juvenile *Ciona* initiate feeding and will begin to concentrate the bacteria within its gut. The stained bacteria are thereafter mostly undetectable anywhere other than the gut of the animal by fluorescent microscopy. This is independent of the type of bacteria used and is shown in Figure 6 where three separate *BacLight* stained bacterial genera (*a. Vibrio, b. Pseudoalteromonas*, and *c. Shewanella*) were exposed to the GF animals. After the desired time of exposure and after the dosing water has been removed and the dishes washed thoroughly, it is possible to remove animals, perform CFU counts, isolate DNA, and determine bacterial abundance (i.e., estimate colonization); RNA isolation can also be used to observe host transcriptional behaviors in response to the bacterial exposures.

## Ethics statement

The research described here was performed on the marine invertebrate, *Ciona intestinalis*, and did not involve human or other vertebrate animals of any form. *Ciona* is not protected by any environmental agency in the United States (US). The collection services (M-Rep, Steve LePage) contracted in this study maintain current permits and licenses for collection and distribution of marine invertebrates to academic institutions; special permission was not required to collect these animals. Animals were recovered and brought to the laboratory alive and maintained in clean water with aeration. Handling of live animals was in accordance with the guidelines of our academic institutions, and the use of specific bacteria in *Ciona* colonization experiments is approved by the USF Biosafety Committee under protocol, 1199-IA. Animal waste products were disposed of appropriately.

## Author contributions

BL developed the methods and drafted the manuscript; AL generated figures, helped refine the methods, and edited the manuscript; LJD oversaw experiments and edited manuscript.

## Funding

These studies were supported by the National Science Foundation (IOS1456301) to LJD and by a National Science Foundation Graduate Research Fellowship (Award No. 1144244) to BL.

### Conflict of interest statement

The authors declare that the research was conducted in the absence of any commercial or financial relationships that could be construed as a potential conflict of interest.
